# Trends in Depression Cases Among Healthcare Workers During the COVID-19 Pandemic and Associated Risk Factors

**DOI:** 10.1192/j.eurpsy.2026.11021

**Published:** 2026-07-31

**Authors:** A. Gaddour, R. ghezal, I. Kacem, A. chouhane, I. jammeli, M. Bouhoula, M. maoua, H. Kalboussi, O. Elmaalel, S. Chatti, A. Brahem

**Affiliations:** 1 Occupational Medicine Department, Ibn El Jazzar teaching Hospital, Kairouan; 2 Faculty of Medicine of Sousse, Research Laboratory LR19SP03; 3 Family Medicine Department, University of Sousse; 4 Occupational Medicine Department, Farhat Hached Teaching Hospital; 5 Occupational Medicine Department, Sahloul Teaching Hospital, Sousse, Tunisia

## Abstract

**Introduction:**

The COVID-19 pandemic has caused unprecedented stress for healthcare workers (HCWs), exacerbating existing mental health challenges such as depression and anxiety. HCWs have been on the frontlines, facing increased workloads, exposure to infection, and emotional distress due to patient outcomes. This study examines the long-term psychological impact of the pandemic on HCWs, focusing on depression severity and associated risk factors.

**Objectives:**

To evaluate the long-term mental health consequences of the COVID-19 pandemic on HCWs, specifically the prevalence and severity of depression, and to identify key factors contributing to psychological distress

**Methods:**

A retrospective descriptive study was conducted on HCWs in Sousse, Tunisia, who took long-term sick leave due to depression between January 2010 and December 2021. Data from medical records were supplemented with a questionnaire covering pandemic-specific stressors and risk factors.

**Results:**

A total of 650 cases were identified. The mean number of depression cases almost doubled, increasing significantly by 89% after the onset of the pandemic (*p* < 0.01). However, no significant correlation was found between depression severity and the occurrence of COVID-19 itself.Among HCWs, the most affected groups included those working in emergency and critical care units, with a significant association between the number of night shifts and depression severity. Additional risk factors included previous psychiatric conditions, high job strain, and direct exposure to COVID-19 patients

**Conclusions:**

The COVID-19 pandemic has amplified the mental health challenges faced by HCWs, particularly regarding depressive disorders. Targeted psychological support and workplace interventions are crucial to mitigate the long-term effects on this vulnerable population.

**Disclosure of Interest:**

None Declared

